# Prenatal attachment & socio-demographic and clinical factors

**DOI:** 10.1192/j.eurpsy.2021.549

**Published:** 2021-08-13

**Authors:** L.S. Meddouri, S. Bourgou, R. Fakhfakh, D. Bousnina, A. Triki, A. Belhadj

**Affiliations:** 1 Child And Adolescent, Mongi Slim Hospital, La Marsa, Tunisia; 2 Preventive Medecine, Aberahman Mami, ariana, Tunisia; 3 Preventive And Social Medecine, mother infant protection center, ezzouhour, Tunisia; 4 Gynecology And Obstetrics, Mongi Slim Hospital, La Marsa, Tunisia

## Abstract

**Introduction:**

A pregnant woman’s bond with her fetus and the quality of the prenatal attachment can be determined by numerous variables.

**Objectives:**

Determine the socio-demographic and clinical factors’ effect on prenatal attachment.

**Methods:**

We conducted a transversal descriptive study in a first line clinical practice center and in an university gynecology-obstetrics department. The Prenatal Attachment Inventory (PAI) was used to assess maternal-fetal attachment.

**Results:**

For the 125 pregnant women that participated in our study, 99,2% were married with consanguinity for 14,4%. The mean marriage duration was 4 years and 3 months. Women were illiterate in 3,2% and more than the half (54,4 %) were unemployed. On average, the current pregnancy was their second one. Pregnancy was spontaneous in 85,6%, unplanned in 71,2% and not desired in 29,6%. Sex of the fetus was not desired by the mother in 40,8%. Dysgravidia complicated 32% of the pregnancies with hospitalization in 25,6%. Fetal health problems were detected in 7,2%. A psychiatric trouble has been reported by 4% of the pregnancies. The total score of PAI ranged from 27 to 82 in our sample. We found a statistically significant negative correlation between PAI and duration of marriage (p=0,012); PAI and gestation number (p=0,039) ; and a correlation between PAI and the planning of the pregnancy (p=0,030).

**Conclusions:**

Socio-demographic and clinical factors should be taken in consideration while evaluating pregnant women at risk of perinatal psychological difficulties.

**Conflict of interest:**

No significant relationships.

